# Gut microbiota-modulating agents in alcoholic liver disease: Links between host metabolism and gut microbiota

**DOI:** 10.3389/fmed.2022.913842

**Published:** 2022-07-22

**Authors:** Jang Han Jung, Sung-Eun Kim, Ki Tae Suk, Dong Joon Kim

**Affiliations:** ^1^Department of Internal Medicine, Hallym University College of Medicine, Chuncheon, South Korea; ^2^Institute for Liver and Digestive Diseases, Hallym University, Chuncheon, South Korea

**Keywords:** alcoholic liver disease, microbiota, dysbiosis, gut-liver axis, host metabolism, fecal microbial transplant (FMT)

## Abstract

Alcoholic liver disease (ALD) involves a wide spectrum of diseases, including asymptomatic hepatic steatosis, alcoholic hepatitis, hepatic fibrosis, and cirrhosis, which leads to morbidity and mortality and is responsible for 0.9% of global deaths. Alcohol consumption induces bacterial translocation and alteration of the gut microbiota composition. These changes in gut microbiota aggravate hepatic inflammation and fibrosis. Alteration of the gut microbiota leads to a weakened gut barrier and changes host immunity and metabolic function, especially related to bile acid metabolism. Modulation and treatment for the gut microbiota in ALD has been studied using probiotics, prebiotics, synbiotics, and fecal microbial transplantation with meaningful results. In this review, we focused on the interaction between alcohol and gut dysbiosis in ALD. Additionally, treatment approaches for gut dysbiosis, such as abstinence, diet, pro-, pre-, and synbiotics, antibiotics, and fecal microbial transplantation, are covered here under ALD. However, further research through human clinical trials is warranted to evaluate the appropriate gut microbiota-modulating agents for each condition related to ALD.

## Introduction

Recent studies have revealed a close relationship between the gut microbiota and host health. The human intestinal microflora is composed of bacteria, fungi, archaea, and viruses (1). On average, there are approximately 500–1,000 types of gut microorganisms in the human gut, and the total number of organisms comprising the gut microbiota is approximately 100 trillion or more (2). Distinct types of gut microbiota exist at distinct locations of the gastrointestinal tract and are also present in the intestinal mucosa and feces. Millions of microorganisms exist in the oral cavity, but their number is reduced in the small intestine due to various factors, such as the presence of gastric and bile acids, and intestinal motility. The major phyla of microbiota present in the small intestine are Firmicutes and Proteobacteria (3). Because the colon takes a long transit time, the number of gut microbiota increases to millions. Anaerobes are mainly present in the large intestine, and the major phyla are Firmicutes (predominantly Ruminococcaceae and Lachnospiraceae), Bacteroidetes, Actinobacteria, Proteobacteria, and Verrucomicrobia (*Akkermansia*) (4).

The gut microbiota coexists harmoniously with the host while maintaining a mutual relationship. The microorganisms play beneficial roles in the host body, such as maintaining a normal immune system, preventing pathogen colonization, and digesting and absorbing nutrients (5). Over the past decade, an extensive amount of research has been published explaining the close relationship between the diversity of organisms in the human gut microbiome and human health and disease. The gut microbiota also maintains homeostasis in terms of diversity and function to maintain the normal health of the host. Various gut microorganisms have common and overlapping functional properties. Therefore, even if a specific strain among the gut microbiota changes, it is compensated by other strains that produce metabolic substrates and metabolites that can express similar functions related to maintaining homeostasis (6). Recently, as the analysis method for gut microbiota metabolomics has been developed, it has been discovered that the metabolic function on the host is different even between similar strains (7). This means that it is possible to analyze the causal relationship between gut microbiota and host disease, and it is possible to confirm the meaning of the role of gut microbiota in host disease.

The close relationship between the gut microbiota and host can be predicted through anatomical structure. Metabolites produced by the gut microbiota enter the liver *via* portal vein circulation (8). Since substances absorbed in the intestine interact with hepatocytes and immune cells of the liver in the liver sinusoids, the liver is called the largest immune organ. That is, the gut-liver axis represents the bidirectional relationship between the gut and gut microbiota resulting from the interaction of genetically evolved biochemical signals and environmental factors, including effects of the host’s diet. The role of the gut microbiota in liver disease, such as bacterial infection in alcoholic liver disease (ALD) and progressive chronic liver disease, has long been noted (9).

Alcohol-related harm is one of the most common preventable sources of disease worldwide, with 3 million deaths or 5.3% of all global deaths attributable to alcohol (10, 11). Alcoholic injury is multisystemic and adversely affects the quantity and quality of life of affected individuals and their family members (12). ALD is a group of diseases with a diverse spectrum, including alcoholic fatty liver, alcoholic steatohepatitis, and alcoholic liver cirrhosis, and the risk of liver cancer due to cirrhosis also increases (13, 14). Although there are no data on the exact prevalence of ALD worldwide, it was reported that total alcohol per capita consumption in the world’s population over 15 years of age increased compared to the past according to 2018 data of WHO (11). Since the close relationship between alcohol use disorders and ALD is well known (15), it can be expected that ALD will also increase worldwide. In addition, in the case of alcohol-associated cirrhosis, which is a severe disease among ALDs, the global prevalence of compensated cirrhosis did not differ significantly between 1990 and 2017 (290 per 100,000 in 1990 vs. 288 per 100,000 in 2017), but that of decompensated cirrhosis was increased (25 per 100,000 in 1990 vs. 30 per 100,000 in 2017) (16). Through above global data, the clinical and socioeconomic significance of alcoholic liver disease could be regarded as it is increasing. Alcohol consumption that affects the occurrence of ALD corresponds to more than 3 drinks per day for men and more than 2 drinks per day for women or binge drinking (more than 5 drinks for men and more than 4 drinks for women over 2 h) (17). However, only approximately 15–20% of drinkers develop ALD (18). This is believed to be due to the pathological mechanisms of ALD, which includes a complex matrix of interactions between the direct effect of alcohol and the toxic metabolites produced by various cells in the liver (19). Lipopolysaccharide (LPS), one of the important factors in the pathophysiology of ALD, induces hepatic steatosis and promotes inflammation (20). The source of this LPS is known to be the gut microbiota in which alcohol-induced barrier permeability is impaired. In other words, it can be interpreted that the influence of the gut microbiota on the pathological mechanisms of ALD may be significant. Recently, trials to improve chronic liver disease by correcting the gut microbiota have been reported (21, 22). In this review, changes in the gut microbiota caused by alcohol and the role of the gut microbiota in the pathophysiology of ALD are summarized. Additionally, studies using the gut microbiota in methods for the improvement of ALD are reviewed and the role of gut microbiota as a treatment in ALD is discussed.

## Gut dysbiosis and alcoholic liver disease

### Gut dysbiosis

The use of the word “dysbiosis” is not an unfamiliar or novel word. Its first use was by a novelist named Elliott Furney. At the time, dysbiosis meant “difficult living,” which is very different from the meaning used in gut microbiota recently (23). The first paper to be used in the study of the gut microbiome was written by Scheunert (24). In this study, it was argued that dysbiosis of the gut microbiota was associated with disease in horses. Haenel was a researcher who emphasized the negative condition, i.e., dysbiosis, which means imbalance, compared to “eubiosis,” a condition that positively affects the host. Haenel analyzed the gut microbiota by studying human intestinal substances and feces (25, 26). However, the criteria to define dysbiosis are still ambiguous because the term can mean an increase or decrease in the total number of microorganisms present in the intestine or an increase or decrease in gut microbial diversity. In each study, dysbiosis has been used to define a change in the composition of the gut microbiota, disturbance and loss of diversity (27), or a condition that negatively affects the host through an imbalance in the gut microbiota (28). In some cases, dysbiosis has been defined as a condition in which the composition of a specific gut microbiota was changed (29). The most commonly used definition of dysbiosis is imbalance (30). To define the meaning of imbalance as a change in the distribution of gut microbiota that leads to negative consequences, the definition of the meaning of balanced or homeostatic must be clear (31). Diversity is always mentioned when discussing the state of a balanced gut microbiota. The composition of the gut microbiota varies between individuals (32). Considering the numerous factors that affect the composition of the gut microbiome, the diversity between individuals is easily understood. Although there are studies that suggest the distribution of intestinal microbes starts from the fetus by the discovery of bacterial DNA or bacterial products in amniotic fluid or placenta (33), the evidence is not yet sufficient, so it is assumed that the intestines before birth are in a sterile state (34, 35). As they begin oral diets after birth, the gut microbiota begins to colonize rapidly. In the process of gut microbiota colonization, the genetic background of the host plays a vital role (36). Pioneer strains successfully colonize the gut to form the gut microbiota, regulating host gene expression and influencing the later diversification of gut microbiota community. In addition, environmental factors such as age, diet, stress, and medications have a significant impact on the composition of the gut microbiota (37). It can be confirmed that the role of the host in maintaining the gut microbiota is as important as the gut microbiota affecting the metabolism of the host. However, some researchers have suggested that the diversity of the gut microbiome may not always indicate a healthy state (38, 39). As mentioned above, and the meaning of dysbiosis is ambiguous, for example, one study evaluated the dysbiosis status using the relative abundance ratio of *Faecalibacterium prausnitzii*, known as an anti-inflammatory strain, and *Escherichia coli*, which predominates in the inflamed intestine (40). In this study, dysbiosis meant the distribution of specific gut microorganisms rather than a change in diversity. This was an effort to define dysbiosis through quantitative criteria. However, recent studies with large human samples have shown that the link between many medical conditions and changes in the gut microbiota is lower than previously expected (41). In addition, other studies have shown that certain gut microorganisms observed in dysbiosis have a positive effect on the health of the host (42). Therefore, it is not appropriate to define dysbiosis as a measure of the proportion of specific gut microorganisms. Currently, the term dysbiosis used in many studies is still difficult to define, and more scientific evidence is needed (30).

### Gut dysbiosis in alcoholic liver disease

Prolonged alcohol intake leads to overgrowth of gut microbiota in laboratory animals and humans. In the case of intragastric alcohol feeding in rats, overgrowth of gut microbiota in the proximal small intestine and large intestine was observed (43, 44). The same results were confirmed in humans. In one study that observed changes in intestinal microflora using culture-based methods, it was confirmed that alcohol caused overgrowth of aerobic and anaerobic bacteria of the jejunum (45). Other studies have also reported microbial overgrowth in the small intestine in patients with alcoholic cirrhosis and moderate alcohol intake (46, 47). Furthermore, it was confirmed that a significant relationship exists between the severity of alcoholic liver cirrhosis and the overgrowth of microorganisms in the small intestine.

**FIGURE 1:**
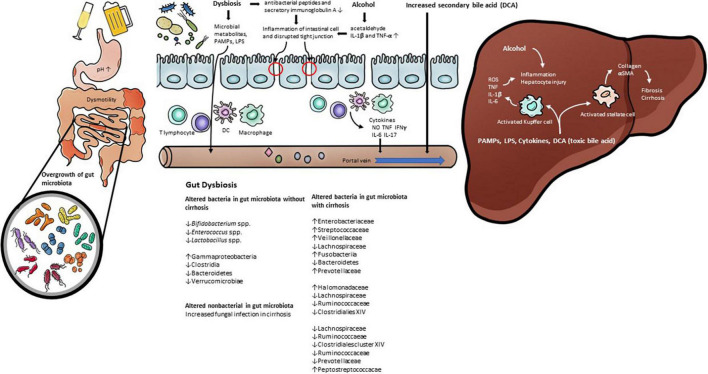
The pathophysiology of gut dysbiosis in alcoholic liver disease. Prolonged alcohol intake leads to change gut permeability and gut microbiota. Alcohol consumption increase inflammatory cytokines such as Il-1β. Gut dysbiosis induce pathological bacterial translocation produced reactive oxygen species (ROS), inducible nitric oxide synthase (iNOS), pathogen-associated molecular patterns (PAMP), such as LPS, TLR4. In addition, alcohol and gut dysbiosis affects bile acid metabolism that has a negative effect on alcoholic liver disease.

Alcohol consumption also induces changes in the gut microbiota composition. A decrease in the phylum Firmicutes and the genus *Lactobacillus* spp. within the phylum Firmicutes was observed in the intestines of mice injected with alcohol into the gastrointestinal tract (43, 44, 48), and the following were observed to increase: *Enterococcus* spp. (phylum Firmicutes), *Akkermansia muciniphila* (phylum Verrucomicrobia), *Corynebacterium* spp. (phylum Actinobacteria), and *Alcaligenes* spp. (phylum Proteobacteria) (43, 44, 48, 49). In humans, a decrease in Bacteroidetes and an increase in Proteobacteria were observed in drinkers with or without ALD compared to healthy controls (50). It was also observed that *Lachnospiraceae, Ruminococcaceae*, and Clostridiales Family XIV *Incertae Sedis* were decreased in patients with alcohol-related cirrhosis (51–54). The *Enterobacteriaceae*, including their prominent genus *Escherichia coli*, were observed to increase (51–55). Bajaj et al. proposed the cirrhosis dysbiosis ratio (CDR) to define dysbiosis in cirrhosis patients (53). The CDR is the ratio of the measures of the autochthonous bacteria *Lachnospiraceae, Ruminococcaceae*, and Clostridiales Family XIV *Incertae Sedis*, which are known to play a positive role in the host, and the measures of *Enterobacteriaceae* and *Bacteroidaceae*, which are known as potential pathogenic species. As seen from the changes in the intestinal microflora observed in previous studies, it was confirmed that, among patients with liver cirrhosis, the CDR was lower in alcohol-induced cirrhosis than in cases of other types of cirrhosis, which resulted from the increase in gram-negative *Enterobacteriaceae* and was reported as a related result. Alcohol consumption induces a decrease in the “good” symbiotic *Lactobacillus* spp. and an increase in “bad” strains of *Enterobacteriaceae.* However, even if the changes in the intestinal microflora caused by alcohol are recovered due to abstinence, it does not improve the intestinal permeability (56). Although this study found that changes in the distribution of gut microbiota play a key role in the host’s metabolic process, it also confirmed that more studies are needed on the role of gut microbiota in improving the altered metabolic process. Table 1 summarizes the studies that observed changes in the gut microbiota in human ALD. In addition, Figure 1 demonstrates the pathophysiology of gut dysbiosis in alcoholic liver disease.

**TABLE 1:** Altered gut microbiota in alcoholic liver disease.

Study	Participant (number)	Methodology	Altered gut microbiota in alcoholic liver disease group
**Alcoholic liver disease without cirrhosis**			
Bode et al. (45)	Alcoholic patients (27) vs. Hospitalized control patients (13)	Aerobic and anaerobic bacterial culture of jejunum aspirates	↑Gram-negative anaerobic bacteria ↑Endospore-forming rods ↑Coliform microorganisms
Kirpich et al. (57)	Alcoholic patients (66) vs. Healthy control (24)	Quantitative culturing of stool samples	↓*Bifidobacterium* spp. ↓*Enterococcus* spp. ↓*Lactobacillus* spp.
Mutlu et al. (50)	Alcoholics with and without alcoholic liver disease (47) vs. Healthy control (18)	16S rRNA gene amplicon sequencing of sigmoid mucosa biopsies	↑Proteobacteria ↑Gammaproteobacteria Firmicutes ↑Bacilli & ↓Clostridia ↓Bacteroidetes ↓Bacteroidetes class Verrucomicrobia ↓Verrucomicrobiae
Leclercq et al. (56)	Alcohol dependent patients before alcohol abstinence (60) vs. Alcohol dependent patients after alcohol abstinence (44)	16S rRNA gene amplicon sequencing and quantitative real-time PCR of stool samples	↑*Bifidobacterium* spp. ↑*Lactobacillus* spp. ↓*Holdemania* spp.
**Alcoholic cirrhosis**			
Chen et al. (51)	Alcoholic cirrhosis (12) vs. Hepatitis B cirrhosis (24) vs. Healthy control (24)	16s rRNA gene amplicon sequencing of stool samples	↑Proteobacteria ↑Gammaproteobacteria ↑Enterobacteriaceae Firmicutes ↑Bacilli ↓Streptococcaceae Clostridia ↑Veillonellaceae and ↓Lachnospiraceae ↓Fusobacteriota ↓Fusobacteriia ↓Bacteroidetes Bacteroidota ↑Prevotellaceae
Bajaj et al. (53)	Alcoholic and non-alcoholic cirrhosis (219) vs. Healthy control (25)	16s rRNA gene amplicon sequencing of stool samples	↑Enterobacteriaceae ↑Halomonadaceae ↓Lachnospiraceae ↓Ruminococcaceae ↓Clostridialies XIV
Tuomisto et al. (58)	Alcoholic cirrhosis (13) vs. Alcoholics without cirrhosis (15) vs. Non-alcoholic control (14)	quantitative real-time PCR of stool samples	↑gram-negative *Bacteroides* spp. ↑gram-negative Enterobactericeae ↑gram-negative *Enterobacter* spp.
Dubinkina et al. (59)	Alcohol dependence syndrome with cirrhosis (27) vs. Alcohol dependence syndrome without cirrhosis (72)	“Shotgun” metagenome analysis of stool samples	↑*Bifidobacterium*: *B. longum, B. dentium, and B. breve* ↑*Streptococcus*: *S. thermophilus and S. mutans* ↑Multiple *Lactobacillus*: *L. salivarius, L. antri, and L. crispatus* ↓*Prevotella* ↓*Paraprevotella* ↓*Alistipes* ↑*Streptococcus constellatus* ↑*Streptococcus salivarius* ↑*Veillonella atypica* ↑*Veillonella dispar* ↑*Veillonella parvula* ↓*Parabacteroide*: *P. distasonis, P. johnsonii, and P. merdae* ↓*Prevotella*: *P. copri and P. disiens* ↓*Clostridium*: *C. asparagiforme, C. methylpentosum, C. saccharolyticum*-like K10, and *C.* sp. L2–50 ↓*Paraprevotella xylaniphila* ↓*Odoribacter splanchnicus* ↓*Phascolarctobacterium* sp. YIT 11841 ↓nine species from the *Bacteroides* genus
Bajaj et al. (60)	Cirrhosis with active drinking (37) vs. Cirrhosis with non-drinking (68) vs. Healthy control (34)	16s rRNA amplicon sequencing of stomach, terminal ileum, and colon biopsies and stool samples	↓Lachnospiraceae ↓Ruminococcaeae ↓Clostridiales cluster XIV ↓Ruminococcaceae ↓Prevotellaceae ↑Peptostreptococcacae ↑Proteobacteria (Enterobacteriaceae) ↓Bacteroidaceae

### Non-bacterial gut microbiota in alcoholic liver disease

Members of the gut microbiota include bacteria as well as fungi, archaea, and viruses. Evidence has been reported that alcohol induces changes not only in bacteria but also in non-bacterial gut microorganisms. Fungal infection adversely affects mortality in patients with alcoholic cirrhosis, which is attributed to a shift in the post antibiotic gut microbiota in patients with alcoholic cirrhosis susceptible to bacterial infection (61). In addition, in animal experiments in the ALD model, fungal overgrowth was observed due to alcohol injection (62). In particular, it was observed that hepatic damage worsened due to an increase in *Candida* spp. It is known that intrahepatic damage caused by fungi occurs because IL-1β is activated after β-glucan of the fungal cell wall is attached to CLEC7A (C-type lectin-like receptor) expressed on the surface of Kupffer cells. Alcohol-induced changes in the non-bacterial gut microbiota are also a principal factor in host metabolic processes. Although studies on non-bacterial gut microbiota are still lacking compared to those on bacteria, biochemical crosstalk between bacteria and non-bacterial gut microorganisms is also expected to play a significant role.

### The pathophysiology of alcoholic liver disease with gut dysbiosis

The gut-liver axis is a major pathway in the development and progression of ALD. Substances produced by intestinal microbes, nutrients absorbed through the intestine, and various substances, including bile acid, flow into the liver through the portal vein and induce various metabolic changes. In a healthy state, the intestinal barrier functions to prevent toxic substances produced by gut microorganisms from flowing into the body through portal flow. The intestinal barrier contains symbiotic microorganisms, the mucosal layer contains secretory immunoglobulin A and antimicrobial peptides, the epithelial intestinal layer consists of tight junctions, and the lamina propria layer in which innate and adaptive immune cells exist (63). The gut-vascular barrier also prevents translocation of gut microorganisms (64). However, many of these intestinal barriers are disrupted by alcohol (65).

#### Cofactors of gut dysbiosis in alcoholic liver disease

Alcohol changes the intestinal environment and promotes an imbalance in the gut microbiota. It is known that drinking alcohol causes disturbances in intestinal motility (66). A decrease in intestinal motility is also observed in patients with cirrhosis, accompanied by overgrowth of gut microbiota (67). There is a case in which the overgrowth of the gut microbiota was improved as intestinal motility was improved in patients with cirrhosis (68). Based on this, it can be considered that decreased intestinal motility caused by alcohol promoted the imbalance of gut microbiota.

Alcohol also decreases gastric acid secretion (69). It is known that hypochlorhydria occurring in liver cirrhosis patients is related to the overgrowth of microorganisms in the small intestine (70), so it can be considered that hypochlorhydria induced by alcohol will also affect the imbalance of intestinal microorganisms.

It has been confirmed through animal experiments that changes in the intestinal innate immune system, which play a key role in the composition of the gut microbiota, can be induced by chronic drinking (43, 44).

#### Consequences of gut dysbiosis in alcoholic liver disease

An imbalance in the gut microbiota caused by alcohol and changes in the intestinal environment induce pathological bacterial translocation. Pathological bacterial translocation refers to the migration of viable bacteria or microbial products to the extraintestinal organs, which is a well-known cause of liver tissue damage (71). For pathological bacterial translocation to occur, intestinal permeability must be increased due to weakening of the intestinal barrier (72, 73). Intestinal epithelial barrier damage is induced by the alcohol metabolite acetaldehyde (74). Reactive oxygen species (ROS) are generated by cytochrome p450 2E1 (CYP2E1), and alcohol-induced liver damage is induced by the increase in CYP2E1 by alcohol (75). Intestinal CYP2E1 also induces an increase in intestinal permeability (76). Alcohol increases intestinal permeability by increasing proinflammatory mediators such as IL-1β and TNF-α in the intestine, leading to intestinal inflammation (77, 78). There have also been studies confirming that inducible nitric oxide synthase (iNOS), a factor that increases intestinal permeability, is a downstream intracellular signaling molecule of TNF-receptor 1 caused by chronic alcohol consumption (78, 79).

Gut dysbiosis plays a key role in the process of intestinal inflammation associated with increased intestinal permeability. This was demonstrated in a study confirming the improvement of intestinal bacterial overgrowth, intestinal inflammation, and intestinal permeability using non-absorbable antibiotics (78). In the group with increased gut permeability among drinkers, a decrease in the distribution of certain gut microbes, such as *Bifidobacterium* spp., Clostridiales Family XIV *Incertae sedis*, and *Ruminococcaceae*, was observed. However, no change was observed in the distribution of gut microbes listed above in the group of drinkers who did not have increased gut permeability (56). This means that there are other factors that affect intestinal permeability in addition to gut dysbiosis, and several factors listed above may have had an influence. One of the main causes of alcoholic liver damage caused by gut microbiota is the translocation of LPS, an important component of the outer membrane of gram-negative bacteria (80). An increase in plasma LPS is observed in cirrhosis as well as ALD (53, 72). The degree of endotoxemia in cirrhosis is related to the degree of liver damage (81), and in particular, endotoxemia is more intense in alcoholic cirrhosis than in other causes of cirrhosis (53). Pathogen-associated molecular patterns (PAMPs), such as LPS, bind to toll-like receptor 4 (TLR4) in the liver and activate immune cells by an intracellular downstream signaling cascade (82). Among the immune cells in the liver, Kupffer cells play an important role in the pathogenesis of ALD (83). Additionally, oxidative stress caused by ethanol and acetaldehyde, a metabolite of alcohol, activate hepatic stellate cells by endotoxin (84). Activation of hepatic stellate cells by TLR4 signaling is required for liver steatosis and inflammation, as well as for fibrosis processes (85).

Bile acid is a representative substance corresponding to the circulation of the gut-liver axis and induces antimicrobial molecules by activating farnesoid X receptor (FXR) of intestinal epithelial cells (86). In patients with cirrhosis, a decrease in bile flow occurs, resulting in overgrowth of gut microbiota (87). The gut microbiota also plays an important role in bile acid metabolism, such as the deconjugation of conjugated bile acids and the conversion of primary bile acids to secondary bile acids (88). Bile salt hydrolase (BSH), which deconjugates bile acid conjugated with glycine and taurine, showed high activity in the gram-positive bacteria *Lactobacillus, Enterococcus, Bacteroides*, and *Clostridium*. *Clostridium scindens, Clostridium hylemonase, Clostridium hiranonis*, and *Clostridium sordellii* are known to be intestinal microorganisms that change primary bile acids to secondary bile acids (89). Alcohol also affects bile acid metabolism, since it is known to stimulate the synthesis of bile acid (90). The pathological mechanism of this phenomenon is known to be that alcohol induces bile acid synthesis through activation of hepatic cannabinoid receptor type 1 and cyclic adenosine monophosphate (cAMP)-responsive element-binding protein H (CREBH) (91). Serum-conjugated deoxycholic acid (DCA) and elevations of total and secondary bile acid in feces were observed in patients with alcoholic cirrhosis who continued drinking compared to patients with non-alcoholic cirrhosis or alcoholic liver cirrhosis who stopped drinking (92). DCA increased by alcohol and gut microbiota activates various cell-signaling pathways (EGFR, AKT, ERK 1/2, PKC, β-catenin, Cox-2), leading to inflammatory NF-κB and proinflammatory cytokine production (93). Through the results of a study in which an increase in the gram-negative strain, mainly Firmicutes, was observed in rats fed an elevated level of cholic acid, it was found that changes in bile acid are a factor inducing changes in the gut microbiota (94). Although no significant changes in gut microbiota were observed in patients with cirrhosis who continued to drink, an increase in *Veillonellaceae* of the phylum Firmicutes and a decrease in *Bacteroidaceae* and *Porphyromonadaceae* of the phylum Bacteroidetes were observed (92). Through this, it can be considered that the change in bile acid is one of the factors that induces the change in gut microbiota.

## Gut microbiota modulating therapies in alcoholic liver disease

**FIGURE 2:**
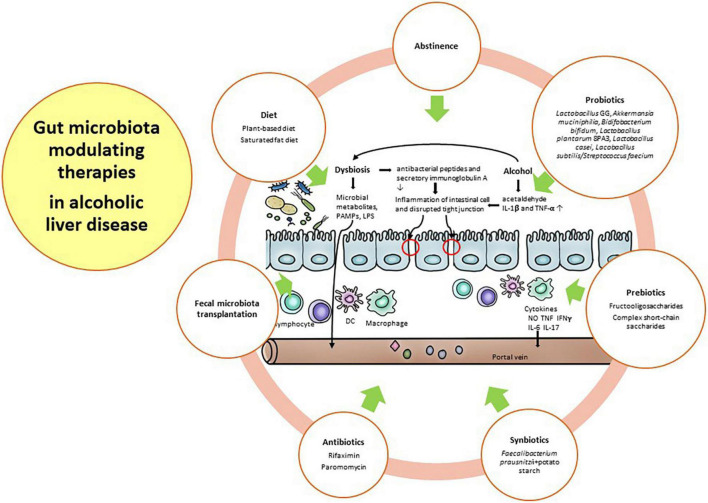
Gut microbiota modulating therapies in alcoholic liver disease.

Alcohol consumption is well known to induce gut dysbiosis, such as *Bifidobacterium*, Clostridium XIV *Incertae Sedis*, and *Ruminococcaceae*, compared with healthy subjects with a weakened gut barrier (56). With prolonged and harmful alcohol consumption, microbial diversity is further decreased, and pathogenic bacteria, such as *Enterobacteriaceae* and *Enterococcaceae*, are further increased. Gut dysbiosis in ALD is relatively more important than other etiologies because alcohol has direct toxicity to both the gut barrier and the gut microbiome before the onset of chronic liver disease (95–97). Therefore, the restoration of the gut barrier and healthy gut microbiome along with abstinence is a major therapeutic target in ALD. For these restorations, diet, antibiotics, probiotics, prebiotics, fecal microbiota transplantation (FMT), and other future strategies will be discussed in this section. Figure 2 depicted the gut microbiota modulating therapies in alcoholic liver disease.

### Abstinence

Abstinence is an important intervention for gut dysbiosis in ALD. Mutlu et al. reported alterations in the mucosal-associated colonic microbiome in only 31% of alcohol-dependent subjects, indicating that not all alcoholics had gut dysbiosis (50). In addition, both actively drinking subjects and sober alcoholic subjects with gut dysbiosis showed incomplete recovery of the gut microbiota after 3 weeks of abstinence, suggesting long-lasting gut dysbiosis in these subjects (50). Interestingly, Leclercq et al. demonstrated that *Lactobacillus* spp. and *Bifidobacterium* spp., as well as the family *Ruminococcaceae*, increased during alcohol abstinence (56). These bacteria, known to have a beneficial impact on gut barrier function (98), could contribute to the recovery of intestinal permeability after alcohol abstinence (56). Ames et al. also demonstrated rapid changes in the gut microbiome following abstinence. They suggest that abstinence affects the recovery of gut dysbiosis, which prevents further organ damage and potentially maintains sobriety (99).

### Diet

Diet may be another major factor in ALD, with the potential to either improve or aggravate underlying disease. In fact, the gut microbiota can be changed within a day by a specific diet, but this effect can be weakened 2 days after cessation of the diet (100). An animal-based diet could alter the gut microbiota, increasing the abundance of bile-tolerant organisms, such as *Alistipes, Bilophilia*, and *Bacteroides*, and decreasing the abundance of Firmicutes, which metabolize dietary plant polysaccharides. This study demonstrated that an animal-based diet could be associated with alterations in fecal bile acid profiles (100). A recent study reported that high-fat diets induced an increase in enteric DCA concentration. This secondary bile acid, as the product of gut microbial metabolism, could inhibit the growth of Bacteroidetes and Firmicutes and promote liver cancer (101). Interestingly, a plant-based diet leads to an increase in the concentration of short-chain fatty acids (SCFAs), such as butyrate and acetate, on the same day (100). Butyrate maintains the gut barrier and provides an energy resource for enterocytes (102). Chronic alcohol intake results in gut dysbiosis characterized by a reduction in the SCFA-producing gut microbiome, such as *Lachnospiraceae* and *Ruminococcaceae* (51, 54, 59, 103–105). Alcohol and dietary fat affect the pathogenesis of ALD. Indeed, the protective effect of dietary saturated fat (SF) and the harmful effect of dietary unsaturated fat (USF) have been well documented in animal models of ALD (106–109). Moreover, epidemiological data showed that dietary intake of SF is associated with lower mortality, whereas dietary intake of USF is associated with higher mortality in patients with alcoholic cirrhosis (110). Kirpich et al. demonstrated that a USF diet (corn oil enriched) exacerbated ethanol-induced endotoxemia and worsened liver disease, whereas an SF diet enriched in medium chain triglycerides (111), the USF diet, induced changes in the gut microbiota with a reduction in Bacteroidetes and enrichment in Proteobacteria and Actinobacteria. This study suggested the importance of dietary factors in ALD potentially by manipulating the gut microbiota. Finally, gut dysbiosis has evolved as a major factor in ALD. Alcohol alters not only the gut microbiome but also the intestinal barrier and might affect various other intestinal functions, such as mucosal immunity. Therefore, therapeutic approaches such as probiotics, prebiotics, synbiotics, antibiotics, and FMT may have the potential to influence and correct gut dysbiosis, which will be discussed in the next section (112).

### Probiotics, prebiotics, synbiotics, and antibiotics

Foods and supplements that may well have exhibited prebiotic or probiotic properties have been used empirically in health maintenance as well as in the treatment of gastrointestinal diseases. Recently, this unregulated and over-the-counter market in supplements of prebiotics and probiotics has begun to attract the inspection of the scientific community and regulatory authorities. The biological effects of these substances affecting gut microbiota are being investigated, albeit too slowly, and various clinical studies of their impact in human diseases are beginning to emerge (113). The Food and Agricultural Organization of the World Health Organization defines a probiotic as being “live microorganisms which when administered in adequate amounts confer a health benefit on the host.” Another- panel of experts convened by the International Scientific Association for Probiotics and Prebiotics (ISAPP) suggested recently that the term probiotic listed 4 categories of compounds or products (live or active cultures, probiotics in food or supplements without a health claim, probiotics in food or supplements with a specific health claim, and probiotic drug) (114). ISAPP defined prebiotics as “a substrate that is selectively utilized by host microorganisms conferring a health benefit (115). In 2004, the definition of prebiotics required (1) resistance to gastric acidity and hydrolysis by enzymes and gastrointestinal absorption; (2) fermentation by gut microbiota; and (3) selective stimulation of the growth and/or activity of gut bacteria (116). Subsequently, the criterion of selective fermentation was removed. In addition, the definition limits prebiotics to interact with the gut microbiota, excluding extraintestinal organs such as the skin and vagina (115). Prebiotics, such as fermentable, soluble fiber, and inulin, are defined as substances that are helpful in promoting the growth and activity of specific gut microbes that confer a health benefit to the host. For example, fibers, such as cellulose, pectins, and xylans promote the enrichment of various microorganisms in the intestine, and prebiotics, such as fructooligosaccharides and galactooligosaccharides, primarily help to proliferate *Lactobacillus* and *Bifidobacterium*. As its name suggests, synbiotics are a combination of prebiotics and probiotics. The intent is to amplify the advantages of the probiotic as well as promote the growth of indigenous beneficial microbes (117). This section will focus on the clinical importance of substances that modulate the gut microbiota in ALD.

#### Probiotics

Probiotics may regulate gut the microbiota, favoring an anti-inflammatory milieu that contrasts bacterial translocation and endotoxin production and restores gut barrier integrity. The mechanisms by which probiotics exert their effects are largely unknown. Probiotics modulate inflammation, reducing gut pH and competing with pathogens for binding and receptor sites (118, 119). To do this, they must have specific characteristics: (1) resistance to bile, hydrochloric acid, and pancreatic juice to reach the small bowel; (2) ability to tolerate stomach and duodenum conditions; (3) stimulation of the immune system; (4) improvement of intestinal function by adhering to and colonizing the intestinal epithelium; (5) competition with pathogens; and (6) modulation of gut permeability (120). The therapeutic role of probiotics has been demonstrated by several mouse models and few human clinical trials in ALD. In a rat model of ALD, Forsyth et al. demonstrated that *Lactobacillus* GG treatment significantly ameliorated hepatic inflammation and preserved gut barrier function along with decreasing alcohol-induced oxidative stress in the small and large intestines (121). They suggested that alcohol-induced endotoxemia leads to a leaky gut, but *Lactobacillus* GG could reduce endotoxemia due to its ability to improve intestinal permeability and decrease bacterial translocation to the liver, consequently reducing the translocation of LPS (121). Huang et al. demonstrated in a rat model of ALD that probiotics and glutamine notably increased the abundance of *Firmicutes* and decreased the abundance of Actinobacteria, Proteobacteria, and *Porphyromonadaceae* with continued alcohol consumption. They reported that probiotic and glutamine treatments ameliorated ALD *via* the suppression of inflammation and the regulation of the gut microbiota (122). In mouse models of ALD, treatment with *Akkermansia muciniphila*, which promotes mucus thickening and gut barrier function, constitutes 1–4% of the fecal microbiota-prevented hepatic inflammation, steatosis, and neutrophil infiltration (123). This gut microbiome did not have the ability to metabolize ethanol, but it was protective against the disruption of the gut barrier induced by ethanol (123). Compared to studies using animal models of ALD, clinical trials for humans are scarce. Short-term treatment using *Bifidobacterium* and *Lactobacillus* was related to restoration of the normal gut microbiome in ALD (57). Kirpich et al. reported that 5 days of administration of *Bidifobacterium bifidum* and *Lactobacillus plantarum* 8PA3 increased the numbers of both Bifidobacteria and Lactobacilli compared to the standard treatment in alcoholic patients. Additionally, alcoholic patients treated with *Bidifobacterium bifidum* and *Lactobacillus plantarum* 8PA3 had significantly lower AST and ALT levels than those who received standard treatment (57). In an open-label study, administration of *Lacobacillus casei* Shirota restored neutrophil phagocytic ability and reduced endotoxin and TLR 4 responses in patients with compensated alcoholic cirrhosis compared with non-treated patients and healthy controls (124). Because this study did not analyze the change in gut microbiota (124), there is a need to evaluate the association between immune function, including neutrophil function, and alteration of the gut microbiota after probiotic treatment in patients with ALD. Interestingly, Han et al. showed that 7-day oral administration of probiotics (cultured *Lactobacillus subtilis/Streptococcus faecium*) in 117 patients with alcoholic hepatitis (probiotics 60 and placebo 57) leads to restoration of the gut microbiota and a reduction in the levels of TNF-α and LPS, except the level of IL-1β, along with improvement of hepatic inflammation (125). In particular, the LPS level and TNF-α level were significantly decreased in patients with cirrhosis after treatment with probiotics. In addition, significant decreases in *E. coli* and *Enterococci* counts were observed in the probiotics-treated group. Therefore, probiotic treatment can modulate the gut microbiota, and probiotics may also be ideal agents for ALD therapy by reducing the overgrowth of harmful bacteria and restoring the normal gut microbiota.

#### Prebiotics

Prebiotics, such as fermentable, soluble fiber and inulin, are defined as substances that are helpful in promoting the growth and activity of specific gut microbes that confer a health benefit to the host (126). Although we recently recognized the extension of prebiotic effects to other groups, such as *Faecalibacterium prausnitzii* (127), *Anaerostipes* spp. (128), etc., beyond Bifidobacteria and *Lactobacilli*, the prebiotic effect in ALD has been studied only for traditional microbes. Yan et al. demonstrated that fructooligosaccharides, complex short-chain saccharides that cannot be digested by pancreatic and brush border enzymes, improved hepatitis and reduced bacterial overgrowth through partially restoring regenerating islet-derived 3 gamma (Reg3 g) protein levels in a mouse model of ALD (43) and they provided evidence of the beneficial effect of prebiotics for Lactobacillus strains in the same mouse model (43). A recent study showed that pectin treatment restored disrupted gut homeostasis in alcohol-fed mice. In the intestine, pectin protected the loss of mucin-producing goblet cells in the colon of alcohol-fed mice. In addition, pectin restored the level of *Bacteroides* and prevented liver injury (129). Prebiotic treatment is warranted to reveal the positive effect in human clinical trials of ALD. Following these studies, we can recommend the therapy of prebiotics in ALD patients.

#### Synbiotics

Synbiotics, combinations of probiotics and prebiotics that provide fuel for probiotics, have been used as treatments in human disease patients (117). Chiu et al. reported that treatment with synbiotics restored intestinal permeability and increased the abundance of Bifidobacteria and *Lactobacilli* in rat models of ALD (130). Prophylactic supplementation with synbiotics provided benefits in a mouse model of chronic-binge alcohol exposure (131). This study hypothesized that synbiotic treatment affects SCFAs such as acetate, propionate and butyrate. Because ethanol is well known to deplete both butyrate and butyrate-producing bacteria (132), they investigated the effect of synbiotics (*Faecalibacterium prausnitzii*, butyrate-producing commensal bacteria with a butyrate-yielding prebiotic, potato starch) deliberately designed to target SCFA in the intestine and inflammation. Supplementation with synbiotics led to improved hepatic inflammation and steatosis (131). The most recent data concern the protective role of synbiotic supplementation in an alcohol-fed rat model, in which synbiotics may reduce muscle protein degradation markers such as beclin-1, which is speculated to be linked to the restoration of intestinal tight junctions and a decrease in liver injury (133). These results should be studied in human trials before further use.

#### Antibiotics

Although antibiotic treatment is often related to the development and spread of resistant microorganisms, rifaximin treatment is recommended in decompensated cirrhosis for the purpose of preventing hepatic encephalopathy and spontaneous bacterial peritonitis (134, 135). Because antibiotic treatment (polymyxin B and neomycin) led to alleviation of liver injury by selective intestinal decontamination in a rat model of ALD (136), Bode et al. investigated whether the non-absorbable antibiotic paromomycin was effective on endotoxemia in patients with ALD (137), but could not demonstrate any beneficial effect of the treatment. The abovementioned rifaximin induced significant changes in the composition of gut microbiota, including an increase in serum saturated (myristic, caprylic, palmitic, palmitoleic, oleic, and eicosanoic) and unsaturated (linoleic, linolenic, gamma-linolenic, and arachnidonic) fatty acids, reducing the networks centered on *Enterobacteriaceae*, *Porphyromonadaceae*, and *Bacteroidaceae*, and indicating a change from pathogenic to beneficial metabolite linkages (138). In a mouse model of visceral hyperalgesia, Xu et al. demonstrated that rifaximin treatment increased the abundance of *Lactobacillus* in the ileum (139). In addition, rifaximin treatment enhanced the abundance of *Faecalibacterium prausnitzii* in patients with irritable bowel syndrome (140) and the abundance of *Lactobacillus* in patients with various gastrointestinal diseases, including irritable bowel syndrome, Crohn’s disease, ulcerative colitis, diverticular disease, and liver cirrhosis with hepatic encephalopathy (141). A smaller, non-randomized trial investigated the effect of rifaximin treatment for patients with ALD. Kalambokis et al. demonstrated that 4 weeks of rifaximin treatment significantly reduced the levels of endotoxin, IL-6, and TNF-α and improved renal function and systemic hemodynamics in patients with alcoholic cirrhosis and ascites (142), but microbial data were not studied. Therefore, larger and longer-term clinical trials are warranted to reveal the beneficial effect of antibiotics such as rifaximin in ALD.

### Fecal microbiota transplantation

FMT is the administration of fecal material containing distal gut microbiota from a healthy human to a patient with a disease or condition related to gut dysbiosis or an alteration in the normal gut microbiota. The aim of FMT is to treat a disease by restoring the gut microbiota. Therefore, FMT has been conducted in various diseases over the past decades (143). When the gut microbiome of heavy drinking subjects with severe alcoholic hepatitis (AH) is transplanted into germ-free mice fed an ethanol-containing diet, severe hepatic inflammation and weakened intestinal permeability are induced in the mice (95). Interestingly, after transplantation of the gut microbiome in healthy subjects, liver injury was ameliorated, despite ongoing alcohol consumption (95). This study suggested important key messages: (1) in patients with severe AH there is a clear microbiota signature such as increased *Bifidobacteria*, *Streptococcia*, and *Enterobacteria*, with decreased *Clotridium leptum* and *Faecalibacterium prausnitzii*; (2) disease can be transferred from man to mouse, thereby suggesting that certain, inherently harmful gut microorganisms may, indeed, exist (95). After FMT was conducted from alcohol-resistant mice to alcohol-sensitive mice, it prevented steatosis and hepatic inflammation and restored gut dysbiosis (129). In an open-label study of FMT in patients with steroid-resistant AH, 1 week of FMT was effective and safe in enrolled patients and improved liver disease severity at 1 year (144). They reported the coexistence of donor and patient microbiota species at 6–12 months post-FMT along with the study of Li et al. (145). These findings suggest that new gut microbiota species from donors, which are beneficial and less pathogenic, can coexist with preexisting gut microbiota communities in the recipient. In another open-label study of FMT in patients with severe AH, the numbers of surviving patients at the end of 3 months after steroid, nutrition, pentoxifylline, and FMT treatment were 38, 29, 30, and 75% (*p* = 0.036), respectively. In patients treated with FMT, the abundance of Actinobacteria and Firmicutes was higher at baseline, while less pathogenic bacteria, such as *Bacteroides, Parabacteroides*, and *Porphyromonas*, predominated at the end of 1 week after FMT, and *Roseburia* and *Micrococcus* predominated beyond 1-month post-FMT (146). A randomized clinical trial (NCT03091010) comparing FMT of steroid treatment in patients with severe AH showed promising data, with an improvement in the 90-day survival rate in the FMT group compared to the steroid group. Although these data have not yet been published, these encouraging results suggest that FMT would be a potentially effective and safe therapeutic option for AH. However, further clinical trials using FMT are needed in patients with ALD, including AH and alcoholic cirrhosis with/without decompensation.

### Future treatment strategies for modulation of gut microbiota

Select members of the gut microbiota may drive the development and progression of ALD, and other members may exert beneficial and protective effects in the development and progression of ALD. Therefore, we discussed the role of probiotics, prebiotics, synbiotics, antibiotics, and FMT as potential treatment options for ALD. Beyond these therapeutic options, several researchers have recently focused on alterations in bile acid metabolism in the small intestine affecting the gut microbiota as postbiotics. Most of the primary bile acids secreted into the intestine are reabsorbed back into the portal circulation, whereas only 5% of primary bile acids are changed to secondary bile acids by the gut microbiota. Therefore, gut dysbiosis alters bile acid metabolism, aggravates secondary bile acid conversion, and reduces the rate of primary bile acid reabsorption (147). In this respect, several studies have demonstrated a significant increase in secondary bile acids in ALD patients with ongoing active alcohol drinking (60, 92, 95). The gut microbiota and bile acid metabolism interact and modulate each other closely through conjugated bile acids binding FXR, which induces fibroblast growth factor (FGF)-19 to decrease the transcription of CYP7a1 in hepatocytes, thereby limiting *de novo* synthesis of bile acids (148). This leads to inhibition of gut microbial overgrowth and restoration of the gut barrier function (86, 149). An intestine-restricted FXR agonist, fexaramine, reduced hepatic inflammation and steatosis in a mouse model of ALD, improved intestinal inflammation, and restored the intestinal barrier (150). Epicallocatechin-3-gallate, as a treatment for obesity, most likely influenced FXR-regulated activity and enriched *Akkermansia muciniphila* (151).

Bioengineered bacteria have been suggested as another therapeutic option for precisely modulating the gut microbiota. Recently, Hendrikx et al. demonstrated that bioengineered *Lactobacillus reuteri* producing IL-22 led to reduced expression of regenerating family member 3 gamma in intestinal epithelial cells, decreased bacterial translocation, and improved liver injury in an ethanol-induced mouse model (152). In addition to illustrating the potential contribution of the gut microbiota to disease pathogenesis, this study emphasizes the vital significance of intestinal immunity. Several studies are already being conducted, and more are needed, to verify the therapeutic effect of the gut microbiota in ALD, and to more precisely characterize the gut microbiota, metabolome, and host response using different preclinical models and larger clinical trials (Table 2).

**TABLE 2:** Ongoing clinical trials for gut microbiota in alcoholic liver disease.

Intervention/treatment	Patient group	Allocation/Intervention model	Trial status	Trial number
Enteral feeding	Alcoholic hepatitis	Case-only/observational	Active, not recruiting	NCT04544020
Synbiotics (Profermin^®^)	Alcoholic liver disease at least F3 fibrosis	Randomized parallel assignment	Active, not recruiting	NCT03863730
Probiotics (Lacidofil^®^)	Alcoholic hepatitis	Randomized/single group assignment	Unknown	NCT02335632
Probiotics (VSL#3)	Alcoholic liver disease, alcohol use disorder	Randomized parallel assignment	Recruiting	NCT05007470
Rifaximin	Alcoholic hepatitis	Non-randomized/single group assignment	Unknown	NCT02116556
Rifaximin	Alcoholic hepatitis	Randomized parallel assignment	Unknown	NCT02485106
Amoxicillin	Alcoholic hepatitis	Randomized parallel assignment	Completed	NCT02281929
Ciprofloxacin	Alcoholic hepatitis, alcoholic cirrhosis	Randomized parallel assignment	Completed	NCT02326103
Vancomycin, gentamycin, meropenem	Alcoholic hepatitis	Single group assignment	Completed	NCT03157388
Fecal microbial transplantation	Alcoholic hepatitis	Randomized parallel assignment	Completed	NCT02458079
Fecal microbial transplantation	Alcoholic hepatitis	Non-randomized parallel assignment	Unknown	NCT03827772
Fecal microbial transplantation	Alcoholic hepatitis	Randomized parallel assignment	Unknown	NCT03091010
Fecal microbial transplantation	Alcoholic hepatitis	Randomized parallel assignment	Not yet recruiting	NCT05006430
Fecal microbial transplantation	Alcoholic hepatitis	Not applicable/single group assignment	Recruiting	NCT04758806
Fecal microbial transplantation	Alcoholic hepatitis	Randomized parallel assignment	Not yet recruiting	NCT05285592

## Conclusion

Alcohol consumption itself induces the changes in the gut microbiota, weakens the gut barrier, and alters host metabolism and immunity results in development and aggravation of ALD. Although various studies have been conducted to reveal the interactions between host and gut microbiota, a comprehensive understanding about these in ALD is still lacking. Therapeutic approaches to gut microbiota such as probiotics, antibiotics, FMT, bio-engineered bacteria, or intestine-restricted FXR agonist are promising in ALD from a new perspective.

## Author contributions

DK: conceptualization. JJ and S-EK: writing of the original draft. JJ, S-EK, KS, and DK: writing, review, and editing. All authors have read and agreed to the published version of the manuscript.

## Conflict of interest

The authors declare that the research was conducted in the absence of any commercial or financial relationships that could be construed as a potential conflict of interest.

## Publisher’s note

All claims expressed in this article are solely those of the authors and do not necessarily represent those of their affiliated organizations, or those of the publisher, the editors and the reviewers. Any product that may be evaluated in this article, or claim that may be made by its manufacturer, is not guaranteed or endorsed by the publisher.
